# Analysis of influenza and parainfluenza respiratory infection in a private tertiary hospital in São Paulo, Brazil

**DOI:** 10.1186/cc14697

**Published:** 2015-09-28

**Authors:** Frederico P Lomar, Carmen SV Barbas, Gustavo FJ de Matos, Humberto B Bogossian, Telma Anunes

**Affiliations:** 1 Hospital Israelita Albert Einstein, Morumbi, São Paulo, SP, Brazil

## Introduction

There are different viruses that cause respiratory infections. Most of the time it is very difficult to know the causative agent from the respiratory symptoms. In order to identify the possibility of a virus being the causative agent of the respiratory infection and to identify the type of virus that is causing the respiratory symptoms, nasal swabs were collected from patients with symptoms of acute respiratory infection.

## Methods

During a 6-month period (1 February-31 July 2014) 365 nasal swabs were collected from patients with symptomatic respiratory infections admitted to Hospital Israelita Albert Einstein, São Paulo, Brazil for polymerase chain reaction virus detection (CLART Pneumovir) capable of detecting 14 respiratory viruses.

## Results

Almost half of the samples collected tested positive. Specifically, 48.76 % (178/365) of samples were positive for respiratory virus. Syncycial respiratory virus accounted for 19.66 % (35/178), influenza H3N2 for 14.61 % (26/178), parainfluenza virus for 7.3 % (13/178), influenza B virus for 6.18 % (11/178), influenza H1N1 for 1.69 % (3/178) and influenza C for 1.69 % (3/178).

## Conclusion

Respiratory virus is a common cause of respiratory infection requiring hospital admission, rhinovirus being the most prevalent. The identification of the type of respiratory virosis is crucial for virus isolation precautions.

